# Fetal pathology meets clinical genetics – on the value of a comprehensive postmortem examination

**DOI:** 10.1515/medgen-2026-2006

**Published:** 2026-04-16

**Authors:** Susanne Sprung, Martina Messner, BSc, Renate Lunzer , BSc, Irene Mutz-Dehbalaie, Patricia Döttelmayer, Karin Freund-Unsinn, Christine Fauth

**Affiliations:** Tirol Kliniken Innsbruck INNPATH, Institute of Pathology Anichstr. 35 6020 Innsbruck Austria; Medical University of Innsbruck Institute of Human Genetics Peter-Mayr-Str. 1 6020 Innsbruck Austria; Medical University of Innsbruck Institute of Human Genetics Peter-Mayr-Str. 1 6020 Innsbruck Austria; Medical University of Innsbruck Department of Gynecology and Obstetrics Anichstr. 35 6020 Innsbruck Austria; Medical University of Innsbruck Institute of Human Genetics Peter-Mayr-Str. 1 6020 Innsbruck Austria; Medical University of Innsbruck Department of Radiology Innrain 52 6020 Innsbruck Austria; Medical University of Innsbruck Institute of Human Genetics Peter-Mayr-Str. 1 6020 Innsbruck Austria

**Keywords:** fetal pathology, placental pathology, fetal phenotyping, clinical genetics, genome wide sequencing

## Abstract

For a long time, a comprehensive postmortem examination has been the most important investigation in unexplained fetal and neonatal deaths. In recent years, the usefulness of autopsy has been questioned due to the availability of improved prenatal imaging techniques and genome-wide sequencing that allow an early prenatal diagnosis of an increasing number of disorders.

While concordance rates of prenatal ultrasound and postmortem findings are high, fetal autopsy may provide additional information in many cases and allow more accurate counseling of parents regarding the recurrence risk and management in future pregnancies.

In this article, we provide a brief outline of a systematic fetal and placental evaluation by pathology and clinical genetics. Based on selected cases, the advantages of a comprehensive postmortem evaluation will be illustrated with emphasis on its role in quality control of prenatal ultrasound after termination of pregnancy, comprehensive and deep phenotyping, the interpretation of genetic variants and its high educational value.

## Introduction

A comprehensive postmortem examination plays an important role in the investigation of fetal and neonatal deaths [1–3]. If a cause or a diagnosis is found, it may allow accurate counseling of parents regarding the recurrence risk and management in future pregnancies. However, autopsy rates decline and parental consent rates are low. This may be related to personal, cultural and religious beliefs of the parents, the invasiveness of the method, the attitude of clinical practitioners perceiving emotional distress as a barrier to discuss autopsy with the parents, and an overall lack of knowledge regarding the value of autopsy [1, 3, 4].

Due to advances in prenatal imaging techniques and the availability of genome-wide sequencing, an increasing number of fetal malformations and genetic disorders now are diagnosed prenatally [5–10]. Genome-wide sequencing has also been used for fetal postmortem analysis to resolve the etiology of unexplained stillbirths and lethal structural fetal anomalies [11–13]. This approach has been termed “molecular” or “genomic” autopsy and promoted as an alternative to classical autopsy, especially in societies with low autopsy acceptance [11, 13]. In parallel, various techniques for fetal postmortem imaging have been developed which allow a “virtual” autopsy by non-invasive visualization of structural fetal anomalies [2, 3].

In view of these advances, the usefulness of classical autopsy may be questioned [2, 3]. However, on closer inspection, there are some caveats: (1) The sensitivity of prenatal ultrasound critically depends on gestational age, and certain birth defects cannot be detected by prenatal ultrasound. In these cases, fetal autopsy can provide important additional information [2, 14]. (2) Interpretation of genetic data is not always straightforward and depends on detailed information on the fetal phenotype and previous reports on cases with a similar phenotype [15, 16]. (3) Prenatal phenotypes of many disorders are still incompletely characterized and may include severe manifestations of well-known syndromes as well as unknown syndromes with antenatal lethality, which await thorough characterization [17–19].

In the following, some of these aspects will be further discussed and illustrated by examples.

## Systematic postmortem evaluation

### Clinical information relevant to the autopsy

Systematic postmortem evaluation starts with a review of relevant clinical information which includes details on the current pregnancy, prenatal ultrasound findings, results of prenatal genetic tests, an obstetric history with information on previous pregnancies, maternal comorbidities and – if available – relevant medical information on the family history [1].

### External and internal examination of the fetus

Examination of the fetus consists of an external and internal examination. Fetuses are preferably examined without prior fixation, which allows taking samples of viable cells for further genetic analysis if needed.

#### Body measurements

External examination of the fetus includes the recording of body measurements, the assessment of fetal growth and maturation in relation to gestational age, and the degree of maceration.

The minimum dataset of fetal measurements includes body weight (W), crown heel length (CHL), crown rump length (CRL), head circumference (OFC), and foot length (FL).

In severely macerated fetuses, body measurements may be inaccurate as CRL, CHL and especially W and OFC are affected by maceration. In these cases, foot length (if feet are not dried out) or femur length (measured by postmortem X-ray) may help to assess gestational age. In a normal fetus, the femur and foot are of comparable length [20].

Postmortem measurements are then correlated to gestational age (as defined by the time of the last menstrual period and results of the “dating ultrasound scan”) and compared to standard growth charts [20]. Significant discrepancies between postmortem measurements and gestational age can indicate fetal growth problems in non-macerated fetuses and help to determine the date of intrauterine death in severely macerated fetuses.

#### External examination of the fetus for malformations and dysmorphism

External evaluation follows a standardized protocol and is performed from head to toe. The examination includes a meticulous search for external malformations and dysmorphic features with thorough delineation of injuries related to delivery (artefactual abnormalities) [1, 20].

In our department, evaluation is routinely performed by a pathologist and a clinical geneticist. The aim of this joint assessment is to identify dysmorphism and malformations, to name differential diagnoses (if possible), to direct further fetal examinations and to guide genetic analyses.

#### Photographic documentation

External photographs are taken of all body parts. The photo documentation includes a full body front and back view with close-ups of the face (frontal and side view), hands, feet, external genitalia, and anus. Dysmorphism and malformations are documented in detail.

#### Dissection and internal examination

Dissection is performed according to standard protocols [21–23]. Internal examination starts with in situ inspection of the thoracic, abdominal, and retroperitoneal organs assessing their anatomy and topographical relationships. All organs are subsequently removed, weighed individually, and sampled for histology, regardless of macroscopic appearance. In cases with complex internal malformations, organs may be removed en bloc, embedded in paraffin and serially sectioned.

Examination of the central nervous system is performed by a neuropathologist and is mandatory in cases of termination of pregnancy for brain/CNS malformations or termination for syndromic disease without unifying diagnosis [23].

Autopsy findings are documented and summarized in the autopsy report including a discussion of the possible or most likely cause of death [21–23].

#### Placenta and umbilical cord

Examination of the placenta is an integral part of autopsy. Standardized processing of the placenta includes macroscopic inspection, determination of the gross and trimmed net weight (after removal of extraplacental membranes and umbilical cord), and measurement of the placental dimensions (length, width, and thickness; calculation of the placental surface). For histological evaluation, a minimum number of three full-thickness sections are taken from different areas of the placenta. In addition, analysis includes transverse sections of the umbilical cord and fetal membranes with reference to the rupture site and the insertion of the umbilical cord. In cases with early onset pre-eclampsia, additional samples of the basal plate are examined for spiral artery pathology [24, 25]. Placental net weight, surface area, and histology can provide information on placental function.

**Table 1:** External examination of the fetus (modified from [1])

Color and appearance of fetal skin: postmortem lividity, changes associated with maceration (skin discoloration, skin slippage, bulla formation, etc.)
Body proportions (symmetry? proportion/disproportion of rump, head, extremities?)
Head: shape, fontanelles, sutures, hair
Neck: length, abnormalities of nuchal skin (thickening, edema?)
Periorbital region and eyes: eyebrows, eyelashes; spacing, length, and slant of the palpebral fissures; irides (color, coloboma?) and cornea (opacity)
Nose: shape, nasal bridge, nares
Philtrum and mouth: philtrum (length, structure), lips, hard and soft palate (including uvula), gums, tongue (size? lobulation? ankyloglossia?), frenula?
Ears: position, shape, external ear canal
Chest: shape, chest wall (defect?), nipples
Abdomen: distension, abdominal wall (defect?), umbilicus and umbilical cord (insertion at the umbilicus, number of vessels, knots, increased coils)
Genitalia: female: labia and clitoris, opening of the vagina; male: penis, position of meatus, scrotum
Anus: position, patency
Back: neural tube defects, sacral dimples
Extremities: length, symmetry, proportion of segments, pterygia, contractures, number of digits and toes, syndactyly, palmar and plantar creases, clinodactyly, nails

The pathology report includes clinical information, a detailed macroscopic and histological description and a classification according to the Amsterdam system [26, 27].

### Taking samples for genetic analysis

In cases without prior genetic testing and a suspected genetic disorder, samples for genetic analysis from the fetus and placenta are taken at autopsy. The selection of suitable tissues depends on the type of analysis and the time of in utero retention after demise: a longer in utero retention time leads to a lower yield of viable cells and a poorer quality of DNA in fetal tissues [28].

*Conventional karyotyping* relies on viable dividing cells. In non-macerated fetuses, various tissues can be used for karyotyping, like skin, lung, umbilical cord, amnion, and placenta. In severely macerated fetuses, viable cells still can be obtained from unfixed chorionic villi / placenta [29].

Vice versa, for *molecular analysis* (molecular karyotyping, exome or genome sequencing etc.) placental tissue is the most reliable source for high-quality DNA even in macerated fetuses. In non-macerated fetuses, thymus or spleen (both of which are rich in lymphocytes and poor in lytic enzymes) may yield good-quality DNA [28].

There are three caveats regarding the use of placental tissue for genetic analysis: (1) Placental mosaicism, which occurs in about 2 % of pregnancies. (2) Contamination of placental tissue by maternal decidua cells (prior to DNA extraction from placental tissue, any adherent maternal tissue should be carefully removed under a dissecting microscope). (3) Differences in the imprinting pattern of placental and fetal tissues [30]. In cases with a suspected imprinting disorder, DNA from fetal tissues should be preferred.

### Radiographs

A whole-body radiograph is essential in fetuses with suspected skeletal dysplasia and other skeletal abnormalities or limb malformations. Fetal postmortem skeletal radiographs usually include an anterior-posterior and a lateral whole-body projection (“babygram”) [31, 32]. Ossification of the fetal skeleton is a sequential process which starts in the late stages of embryonic development at 8 weeks of gestation. A detailed ossification sequence of the fetal skeleton is provided by Calder and Offiah [32] and in the atlas of Schumacher et al. [33].

Postmortem radiographs can provide a definitive diagnosis in pregnancies terminated for an ultrasound diagnosis of a lethal skeletal dysplasia without prior genetic confirmation and guide genetic testing. Radiographs may also rule out a skeletal dysplasia in unspecified conditions with “shortened long bones”.

## Selected topics and examples

At the Medical University of Innsbruck, approximately 90 fetal autopsies are performed per year. More than one third are from pregnancy losses including miscarriages and unexplained stillbirths, and from pregnancies which were terminated due to severe ultrasound abnormalities but without a prior genetic diagnosis. About 25 % of autopsies are after pregnancy termination for confirmed genetic disorders (~ 17 % chromosomal aberrations, ~ 7–8 % monogenic disorders). Infections and premature rupture of membranes are the reasons for autopsy in about 10–15 % of cases.

The following section addresses selected topics in fetal pathology. Postmortem findings in common and rare genetic disorders, cases with discrepancy between prenatal ultrasound and autopsy, and non-genetic causes of stillbirths are exemplified.

### Chromosomal aberrations

The most frequent chromosomal aberrations in induced abortions and stillbirths are trisomy 21, trisomy 18, monosomy X, triploidy, and trisomy 13. In our own cohort most of these fetuses are from pregnancy terminations after prenatal diagnosis of a chromosomal aberration.

*Trisomy 21*: While prenatal ultrasound findings may be quite suggestive for trisomy 21, the postmortem phenotype of affected fetuses is often less clear. The characteristic facial phenotype in trisomy 21 evolves over time and is rarely seen in previable fetuses. There may be unspecific external findings like mild nuchal edema, and, more typical but not specific, a single transverse palmar crease, clinodactyly/brachymesophalangy of the fifth finger or a sandal gap of toes. According to Kalousek, about 10 % of previable fetuses with Down syndrome have no external abnormalities [34].

**Figure 1:**
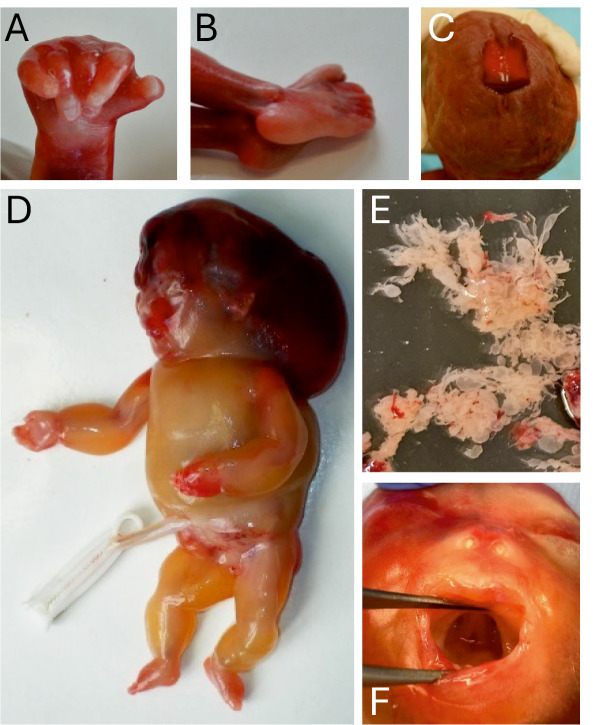
(A-B) Clenched hand and prominent heels in a fetus with trisomy 18. (C) Aplasia cutis congenita (large median scalp defect) in a fetus with trisomy 13. (D) Monosomy X: hydrops fetalis with large cystic hygroma. (E) Hydropic “grape-like” chorionic villi in diandric tiploidy. (F) U-shaped cleft soft palate in a fetus with COL2A1-related achondrogenesis detected on postmortem inspection.

*Trisomy 18*: The clinical phenotype of fetuses with trisomy 18 covers a broad spectrum and ranges from fetal hydrops and various externally visible malformations like omphalocele, neural tube defects and orofacial clefting to growth-retarded fetuses with more subtle anomalies. Like in trisomy 21, the facial phenotype in previable fetuses with trisomy 18 is often unspecific. However, the majority of fetuses with trisomy 18 have characteristic abnormalities of the hands and feet. The typical “clenched hand” with the second and fifth fingers overlapping the third and fourth fingers (figure 1 A), can be seen from the 14^th^ week of gestation onwards [34, 35]. Other frequent findings are club hands (with or without radial aplasia), prominent heels (figure 1 B), and rocker-bottom feet.

*Trisomy 13*: Compared to trisomy 18 and 21, the clinical phenotype in fetuses with trisomy 13 is more severe. Major malformations like holoprosencephaly, broad median cleft lip and palate, severe heart defects, omphalocele and postaxial polydactyly of hands and feet may already be visible on first trimester ultrasound. A striking feature occasionally found in fetuses with trisomy 13 are congenital scalp defects (aplasia cutis congenita) (figure 1 C).

*Monosomy X (Turner syndrome):* Non-mosaic monosomy X (45,X) has a very high in utero lethality with less than 5 % of the affected fetuses surviving until birth [36, 37]. The phenotype of severely affected fetuses markedly differs from that of girls diagnosed with Turner syndrome at birth or in childhood. Early miscarriages and stillborn fetuses with monosomy X typically present with large posterior nuchal cystic hygroma, generalized edema and hydrops fetalis (figure 1 D). Heart disease is common, in particular coarctation of the aorta and hypoplastic left heart syndrome [38].

*Triploidy (3n=69 chromosomes)*: Depending on the parental origin of the additional haploid chromosome set, triploidy is either digynic or diandric. Morphological examination of triploid fetuses and placentas often, but not invariably, allows for distinction between digyny and diandry [39, 40]. Digynic fetuses are severely growth retarded with a marked head-body disproportion (asymmetric growth restriction with relative macrocephaly) and have a very small, nonmolar placenta. In contrast, diandric fetuses tend to be more proportionate, less severely growth retarded and have a large placenta with areas of swollen hydatiform villi (partial mole) (figure 1 E). Common findings in both subtypes of triploidy are 3/4 syndactyly of fingers and 2/3 syndactyly of toes and a wide variety of malformations [41].

### Skeletal dysplasia

The current Nosology of Genetic Skeletal Disorders (2023 revision) lists more than 750 different disorders [42]. Only a subset of them manifest prenatally. Three life-limiting prenatal skeletal disorders account for slightly more than half of all antenatally diagnosed skeletal dysplasias: thanatophoric dysplasia (mainly type 1), the severe perinatal form of osteogenesis imperfecta (COL1A1-/COL1A2-related) and COL2A1-related achondrogenesis (former achondrogenesis type 2, Langer-Saldino) [32, 43, 44].

Lethal skeletal disorders are usually detected by prenatal ultrasound. Lethality is determined by significant narrowing of the thorax with concomitant lung hypoplasia leading to neonatal asphyxia. Other characteristics of lethal skeletal dysplasia are early and severe shortening of long bones (more than 4 SD below the mean), bowing of bones, multiple fractures, absent or reduced ossification of the spine, reduced bone echogenicity and fetal hydrops [43–45].

When ultrasound is suggestive of lethality, couples sometimes opt for termination of pregnancy without prior genetic testing. After termination, a specific diagnosis is best obtained by a dual approach, including detailed phenotyping by postmortem radiographs and (targeted) genetic testing.

In our autopsy cohort, there are on average 2–3 fetuses with a lethal skeletal dysplasia per year. In figure 2 photographs and radiographs of three common lethal skeletal dysplasias are shown.

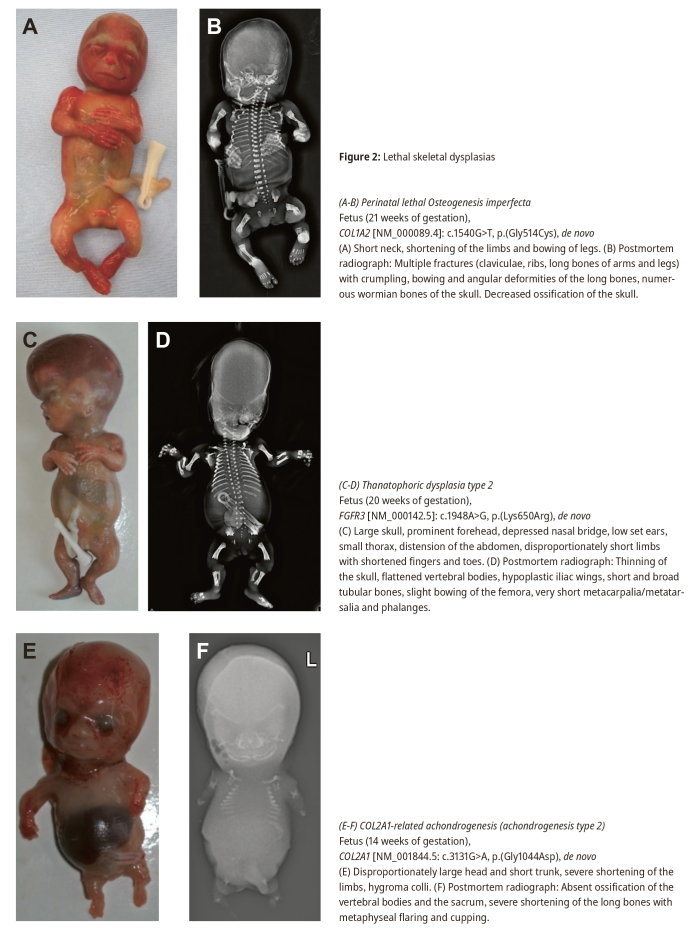



### Severe prenatal manifestations of well-known disorders and novel syndromes

Some disorders cover a broad clinical spectrum of manifestations ranging from mild forms with subtle physical anomalies in children or adults to severe phenotypes with embryonic or fetal lethality. A well-known example of this is Smith-Lemli-Opitz syndrome [46].

However, fetal phenotypes are still incompletely characterized and may include so far undescribed severe manifestations of known disorders as well as novel syndromes with antenatal lethality [18, 19]. Detailed phenotyping is critical for the classification of rare and novel genetic variants and in cases with novel gene-phenotype associations [15, 16, 47].

Two examples are provided below: The first one shows a lethal in utero presentation of a fetus with Smith-Lemli-Opitz syndrome. The second one is an unusual manifestation of severe arthrogryposis in a fetus with a novel genetic variant in the *ACTC1* gene.

#### Example 1: Smith-Lemli-Opitz syndrome

**Figure 3:**
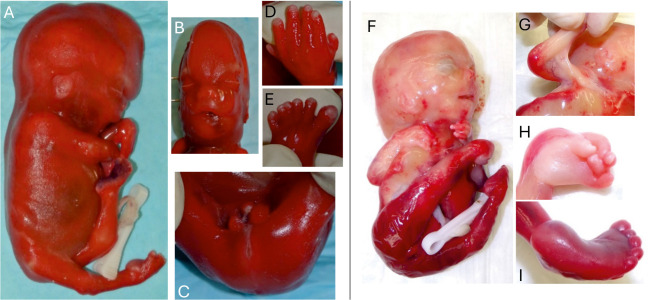
(A-E) Severe manifestation of Smith-Lemli-Opitz syndrome in a male fetus (20 weeks of gestation). (A) Nuchal and skin edema, aberrant hand and leg positioning due to oligo-/anhydramnios. (B) Characteristic facial dysmorphism with bitemporal narrowing, hypertelorism and short nose with anteverted nares. (C) Ambiguous genitalia with bifid scrotum. (D, E) Postaxial polydactyly (right hand and left foot are shown). Syndactyly of toes. (F-I) Female fetus with severe arthrogryposis and a missense variant in *ACTC1* (20 weeks of gestation). (F) Short neck with nuchal edema, multiple joint contractures (hips, elbows, wrists, hands), hyperextension of knees. (G) Pterygium of shoulder and elbow. (H) Arthrogrypotic hand with contractures of fingers and reduced/absent skin creases. (I) Clubfoot.

Figure 3 A-E shows a severe prenatal manifestation of Smith-Lemli-Opitz syndrome in a male fetus homozygous for the common pathogenic splice acceptor variant in intron 8 of the *DHCR7* gene ([NM_001360.3]: c.964–1G>C, p.?). Pregnancy was terminated at 20 weeks of gestation because of hydrops fetalis, cardiomegaly and oligo-/anhydramnios due to agenesis of kidneys. External postmortem inspection revealed typical malformations, including postaxial polydactyly of hands and feet, cleft palate, and ambiguous genitalia. Of note, the characteristic facial phenotype with bitemporal narrowing, hypertelorism, short nose with anteverted nares, microretrognathia, and low-set ears is already visible in the 20^th^ week of gestation.

#### Example 2: Severe fetal arthrogryposis

Figure 3 F-I shows a female fetus with severe arthrogryposis (arthrogryposis multiplex congenita). Pregnancy was terminated at 20 weeks of gestation due to akinesia and hydrops fetalis. On external examination, the fetus had multiple joint contractures (hips, elbows, wrists, and hands), hyperextension of knees, clubfeet, and pterygia of the shoulders and elbows. Autopsy (including a neuropathological examination of the brain) did not reveal any organ malformations. Trio exome analysis identified a heterozygous, previously undescribed de novo missense variant in the *ACTC1* gene affecting a highly conserved amino acid (NM_005159.5: c.1108T>A; p.(Ser370Thr)) [48]. *ACTC1* encodes the primary actin in heart muscle (“alpha cardiac actin”, MIM *102540). Mutations in this gene are linked to cardiomyopathies and other heart anomalies in adults and children (MIM #612098, #613424, #612794) [49]. At first glance, the variant in *ACTC1* seems unlikely to cause fetal arthrogryposis. However, rare variants in *ACTC1* have recently been identified in patients with distal arthrogryposis and in two fetuses with lethal fetal akinesia, multiple contractures and hydrops [50, 51]. Both fetuses carried the heterozygous variant c.1121G>A, p.(Arg374His), which affects a highly conserved amino acid in close proximity to the variant found in our case. Moreover, a previous study on the expression of ACTC1 in fetal cardiac and skeletal muscle has shown that ACTC1 is the predominant sarcomeric actin in *fetal* skeletal muscle. In late pregnancy, ACTC1 is down-regulated and subsequently replaced by ACTA1 (“alpha skeletal actin”), which is the main alpha actin in postnatal skeletal muscle [50, 52]. In view of these findings, a causative role of the *ACTC1* variant c.1108T>A; p.(Ser370Thr) for severe fetal arthrogryposis seems likely.

This case illustrates that fetal phenotypes of monogenic disorders may significantly differ from their well-described postnatal manifestations and may include lethal (allelic) disorders [18, 53]. It also shows that available information on fetal phenotypes is often sparse and may rely on few published cases.

**Figure 4:**
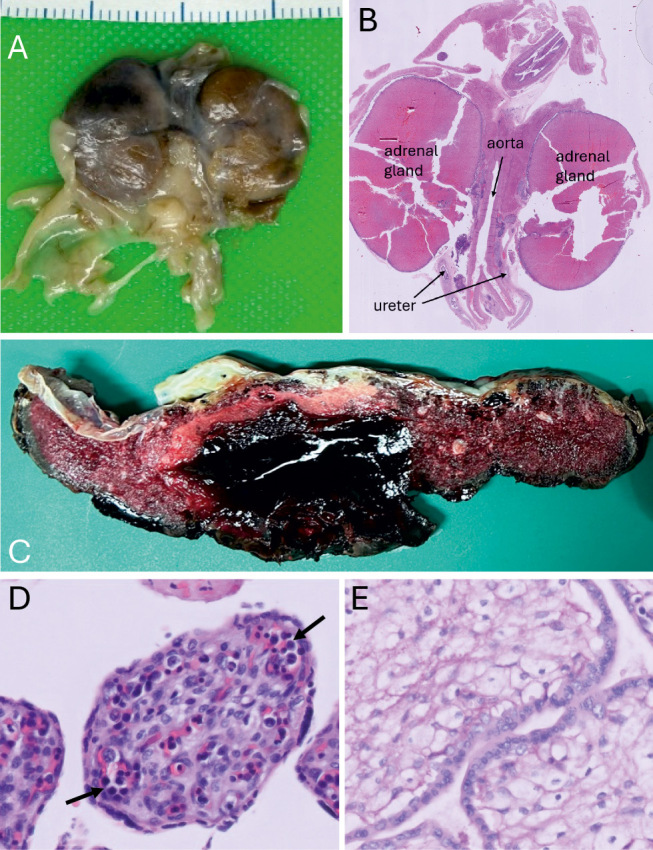
(A) Retroperitoneal organs of a fetus with BOR syndrome and bilateral renal agenesis not detected on prenatal ultrasound (17 weeks of gestation). Note the enlarged “kidney-shaped” adrenal glands. (B) Histological section of the same specimen showing the typical histology of adrenal glands (HE stain). (C) Abruptio placentae with large retroplacental haemorrhage (30 weeks of gestation). Cross-section through the placenta. (D) Chorionic villi, parvovirus B19 infection (HE stain). Note the increased number of fetal erythroblasts with enlarged nuclei (arrows) in the capillaries of chorionic villi. (E) Chorionic villi, granular vacuolated “foamy” Hofbauer cells in MPS VII (PAS stain).

### Discrepancy between prenatal ultrasound findings and autopsy

In non-macerated fetuses, autopsy can provide more detailed and comprehensive information on fetal structural anomalies than prenatal ultrasound. Several studies have shown that fetal autopsy provides additional information in 22–62 % of cases and changes the initial diagnosis in 1–33 % of cases [2, 14]

As a case in point, autopsy findings in a fetus with branchio-oto-renal (BOR) syndrome are shown in figure 4 A and B. While first-trimester ultrasound was unremarkable with normal visualization of both kidneys, a follow-up scan at 17 weeks of gestation revealed oligohydramnios and kidneys appeared small and hyperechogenic. The pregnancy was terminated and autopsy unexpectedly revealed bilateral renal agenesis. The hyperechogenic structures presumed to be kidneys on prenatal ultrasound in fact were enlarged adrenal glands extending into the renal beds.

### Birth defects which are missed by prenatal ultrasound

There are some birth defects which are missed by prenatal ultrasound. Typical examples are clefts of the soft palate, anal atresia, syndactyly of fingers or toes, and abnormalities of the skin. In these cases, postmortem inspection of the fetus and autopsy can add valuable information to the clinical phenotype.

In figure 1 F, a U-shaped cleft of the soft palate in a fetus with COL2A1-related achondrogenesis is shown which was not detected by prenatal ultrasound. Cleft palate is a common finding in type II collagenopathies.

### Diagnoses with placental pathology

Placental anomalies are a direct cause or a major contributor to spontaneous abortions and fetal deaths [54, 55]. According to various studies, pathologic examination of the placenta identifies a cause in 23–65 % of stillbirths [2]. The most frequent placental lesions are maternal placental vascular anomalies (placental infarctions, retroplacental haemorrhage) and infections (bacterial, viral, parasitic) which lead to chorioamnionitis and / or villitis [56, 57].

To illustrate the utility of placental examination, three examples are provided:

#### Vascular anomaly

Figure 4 C shows a large retroplacental haemorrhage in a stillbirth with premature separation of the placenta (abruptio placentae; 30^th^ week of gestation).

#### Infection

Parvovirus B19 infects fetal erythroblasts and may lead to severe anaemia and hydrops fetalis. Typical findings in the placenta are edema, an increase in nucleated red blood cells in the capillaries of chorionic villi and a characteristic morphology of infected erythroblasts with enlarged nuclei and intranuclear inclusions [57, 58]. Figure 4 D shows a histological section of chorionic villi from a pregnancy with Parvovirus B19 infection.

#### Histopathological abnormalities in the placenta indicating fetal storage disorders

Histopathological examination of the placenta can also provide valuable clues to genetic conditions like fetal storage disorders [59, 60]. An example is given in figure 4 E which shows the placenta of a fetus with mucopolysaccharidosis type VII (MPS VII, Sly syndrome). The villus stroma contains granular vacuolated “foamy” Hofbauer cells (placental macrophages) indicating storage of glycosaminoglycans.

## Conclusion and outlook

This article aims to illustrate the usefulness of a thorough external and internal postmortem examination of fetus and placenta. A detailed fetal examination can identify dysmorphism and malformations which are not apparent on prenatal ultrasound, change diagnoses, add valuable information to the clinical phenotype, guide genetic testing, and improve interpretation of genetic variants. A careful examination of the placenta may reveal vascular problems, like maternal placental vascular anomalies, and non-genetic causes for stillbirth, such as infections. Determining the cause of a stillbirth or perinatal death is essential for parents, helps to understand why this had happened and may allow accurate counseling regarding the recurrence risk and management in future pregnancies.

Postmortem examination is a crucial part of deep phenotyping. It contributes to our understanding of phenotypic variation in known disorders and is essential for the characterization of novel disorders [16, 61, 62]. Comprehensive phenotyping is the result of an interdisciplinary effort und includes data from multiple sources (pathology, clinical genetics, prenatal ultrasound and MRI, genetic tests, laboratory findings, etc.) [61]. As every method has its strengths and limitations, different approaches to the fetal phenotype are not mutually exclusive but rather complementary.
